# Selumetinib as a Target Therapy in Progressive Paediatric Low‐Grade Gliomas—Case Series (pLGG)

**DOI:** 10.1111/jpc.70455

**Published:** 2026-06-11

**Authors:** Laura Trapani, Giulia Gortani, Andrea Magnolato, Flora Maria Murru, Elisabetta Cattaruzzi, Laura Diplotti, Paola Michieletto, Silvia Giganti, Davide Zanon, Alessandra Maestro, Valentina Kiren, Egidio Barbi, Irene Bruno

**Affiliations:** ^1^ Clinical Department of Medical Surgical and Health Science, University of Trieste Trieste Italy; ^2^ Institute for Maternal and Child Health IRCCS Burlo Garofolo Trieste Italy

**Keywords:** neurofibromatosis, optic pathway glioma, selumetinib

## Abstract

**Background:**

Optic pathway gliomas (OPGs) occur in 15%–20% of children with neurofibromatosis type 1 (NF1). While smaller gliomas may be only monitored, the current standard of care for symptomatic ones relies on chemotherapy, most commonly carboplatin and vincristine. These drugs can achieve tumour control but are associated with significant toxicity and do not generally restore visual function. Selumetinib, a selective MEK inhibitor, has shown efficacy in reducing NF1‐related plexiform neurofibromas and has emerged as a promising targeted therapy for NF1‐associated low‐grade gliomas.

**Aim:**

To retrospectively evaluate the efficacy and safety of selumetinib in children with NF1‐associated progressive OPGs, with attention to radiological and functional visual outcomes.

**Methods:**

We reviewed three paediatric patients with NF1 and progressive OPGs treated with selumetinib at 25 mg/m^2^/day. In one case, selumetinib was initiated as second‐line after tumour regrowth following chemotherapy; in the other two, it was administered as first‐line under compassionate use. Tumour response was assessed by MRI, visual function with serial ophthalmological evaluations.

**Results:**

All patients showed radiological tumour shrinkage or stabilization with clinically meaningful improvements in visual acuity. One child achieved near‐complete recovery of vision. Treatment was well tolerated: adverse events were mild, predominantly dermatological. No severe systemic toxicities were observed.

**Conclusions:**

While rigorous clinical trial data is still emerging, these preliminary cases suggest selumetinib may be a safe and effective therapeutic option for NF1‐related OPGs, offering significant tumour control with a favourable toxicity profile compared to chemotherapy. Beyond stabilization, its potential to restore visual function represents a major advance, supporting the potential role of MEK inhibition as a first‐ and second‐line treatment strategy.

**Abbreviations**
BCVA: best‐corrected visual acuity
CV: carboplatin plus vincristine
IOP: intraocular pressure
MRI: magnetic resonance imaging
NF1: neurofibromatosis type 1
OCT: optical coherence tomography
OD: oculus dexter (right eye)
OPG: optic pathway gliomas
OS: oculus sinister (left eye)
OU: oculus uterque (both eyes)
RNFL: retinal nerve fibre layer
VEPs: visual evoked potentials

## 1 Introduction and Background

Neurofibromatosis type 1 (NF1) is the most prevalent neurocutaneous disorder (~1:3000 incidence) [1] caused by germline loss‐of‐function mutations in the NF1 tumour suppressor gene, which codes for neurofibromin, a negative regulator of Ras [2]. Tumour formation follows the ‘double‐hit’ model: somatic inactivation of the second NF1 allele leads to constitutive activation of the Raf–MEK–ERK MAPK pathway, driving abnormal cell proliferation. This autosomal dominant syndrome manifests with diverse cutaneous, ocular and neurological symptoms [3].

Optic pathway gliomas (OPGs) are the most common CNS tumours in children with NF1 (15%–20% of patients) and account for 3%–5% of paediatric brain tumours [4]. Most are WHO grade I pilocytic astrocytomas [5], typically arising in early childhood (median diagnosis 4.5 years) [4] and predominantly involving the anterior visual pathway (75%–85% of cases). Clinical courses vary: many are asymptomatic, but 30%–50% progress, often causing irreversible vision loss or endocrine dysfunction from hypothalamic involvement [4]. Young age (< 2 years) and female sex are associated with worse visual outcomes [6]. No validated biomarkers currently exist to identify high‐risk patients.

Asymptomatic NF1‐OPGs are managed with serial MRI and ophthalmologic surveillance due to their unpredictable natural history. Therapy begins with radiological progression or visual decline [7]: a two‐line decrease in visual acuity on standardized assessment warrants referral for treatment.

Carboplatin plus vincristine (CV) remains the standard first‐line chemotherapy for symptomatic or progressive NF1‐OPGs, regardless of the age at presentation, as indicated by the European Cooperative multicentre Study for Children and Adolescents with Low Grade Glioma (SIOP‐LGG2004). According to these guidelines, chemotherapy consists of an induction period with a more intensive schedule from weeks 1 to 10, a less compact phase from weeks 13 to 21 and a prolonged consolidation therapy starting at weeks 25 up to week 81 [8].

The standard regimen achieves meaningful tumour control: in a pivotal pLGG cohort, ORR was 56% and 2‐ and 3‐year PFS rates were 75% and 68%, respectively [9], with similar results reported elsewhere [10, 11]. In a multicentre retrospective review, despite chemotherapy treatment, visual acuity improved in only 32%, while stabilized in 40% and declined in 28% of NF1 [12], while carrying significant risks of hematologic suppression, carboplatin hypersensitivity, ototoxicity and vincristine‐induced neuropathy. Importantly, NF1 patients had a decreased risk of grade 3–4 toxicities compared to non‐NF1 patients in the COG A9952 study [13]. Three second malignant neoplasms occurred in the CV‐NF1 group at a median of 7.8 years [13].

While CV regimen achieves meaningful tumour control, different regimens are used in different parts of the world (e.g., bevacizumab, weekly vinblastine, or three‐weekly vinblastine and carboplatin), all of which have been shown to have reasonably similar toxicity and efficacy [14].

Vinblastine offers an alternative that may reduce neuropathy [15], while multi‐agent regimens like TPCV (thioguanine, procarbazine, lomustine, vincristine) are avoided in NF1 due to the higher risk of secondary malignancies [16]. Surgery and radiation are limited treatments due to tumour infiltration and major long‐term effects on the developing brain [17]. Guidelines strictly recommend that children affected by NF1 with pLGG should not be irradiated unless chemotherapy and surgery have failed.

Selumetinib, a selective MEK1/2 inhibitor, directly targets the dysregulated MAPK cascade in NF1‐associated tumours [2]. Its approval for NF1‐related plexiform neurofibromas came after the SPRINT trial, where 34 out of 50 children achieved durable partial responses and improved pain and quality of life [18].

Following successful use in PNs, the PBTC‐029 multicentre phase II trial assessed selumetinib in children with recurrent, refractory, or progressive pLGGs. Stratum 3 focused on 25 NF1‐associated pLGG patients who had progressed on at least one prior therapy [19].

Stratum 3 results were highly encouraging: 10 of 25 patients (40%) had a sustained partial response to selumetinib and most others had durable stable disease. This high disease control led to a 2‐year PFS of nearly 96% [19], surpassing historical second‐line chemotherapy outcomes.

Given promising prior results, the strong rationale for selumetinib and its known effect on plexiform neurofibromas, we treated three children with pLGGs causing severe visual impairment with selumetinib.

## 2 Materials and Methods

We retrospectively reviewed the clinical records of three paediatric patients with progressive low‐grade glioma (pLGG) treated off‐label with selumetinib (25 mg/m^2^/day). Selumetinib was used as a second‐line therapy in one patient and as first‐line treatment under off‐label use in the other two.

Selumetinib is not routinely utilized as a first‐line therapy: in the last two cases, it was administered under compassionate use following a comprehensive multidisciplinary agreement between paediatric oncologists and rare disease specialists. This decision was heavily driven by the families' explicit desire to pursue targeted therapeutic options with a potentially lower systemic toxicity profile compared to standard chemotherapy.

Inclusion criteria were confirmed progressive pLGG, no prior selumetinib, and clinical need for chemotherapy. Data collected included regimen, dosage, duration, adverse events and outcomes.

In accordance with the schedule outlined in the American Academy of Pediatrics (AAP) guidelines, children with NF1 undergo regular complete ophthalmological assessment, including best‐corrected visual acuity (BCVA), Hirschberg corneal light reflex test, Cover test, Lang I and II stereo test, ocular motility examination, Goldmann intraocular pressure, cycloplegic refractions, slit‐lamp biomicroscopy and dilated‐fundus examinations, visual field test (confrontation test or Goldmann kinetic perimetry, according to age and compliance), visual evoked potentials (VEP), optical coherence tomography (OCT).

The relationship between retinal nerve fibre layer (RNFL) thickness and BCVA in the presence of optic nerve gliomas in NF1 is well established [20], as is the reduction of RNFL thickness in the presence of a glioma (average values below 76 μm), with greater involvement of the superior and inferior sectors. Both OCT and transient pattern VEPs are also useful in predicting future BCVA in untreated gliomas [21]. The importance of monitoring optic nerve function with VEPs in NF1‐related gliomas is emphasized by both Kelly et al. [22] and Tekavčič Pompe et al. [23], although amplitude variations present even in normal subjects can complicate interpretation: pathological values are indicated when the amplitude is < 9 μV. Regarding RNFL, a clinical challenge is represented by its progressive thinning following the appearance of the tumour, which can occur even in the absence of volumetric variations [24].

The use of the logMAR scale allows for the evaluation of visual acuity through a non‐linear logarithmic progression, optimizing diagnostic accuracy, especially in the quantification of low vision.

## 3 Case Presentations

**FIGURE 1:**
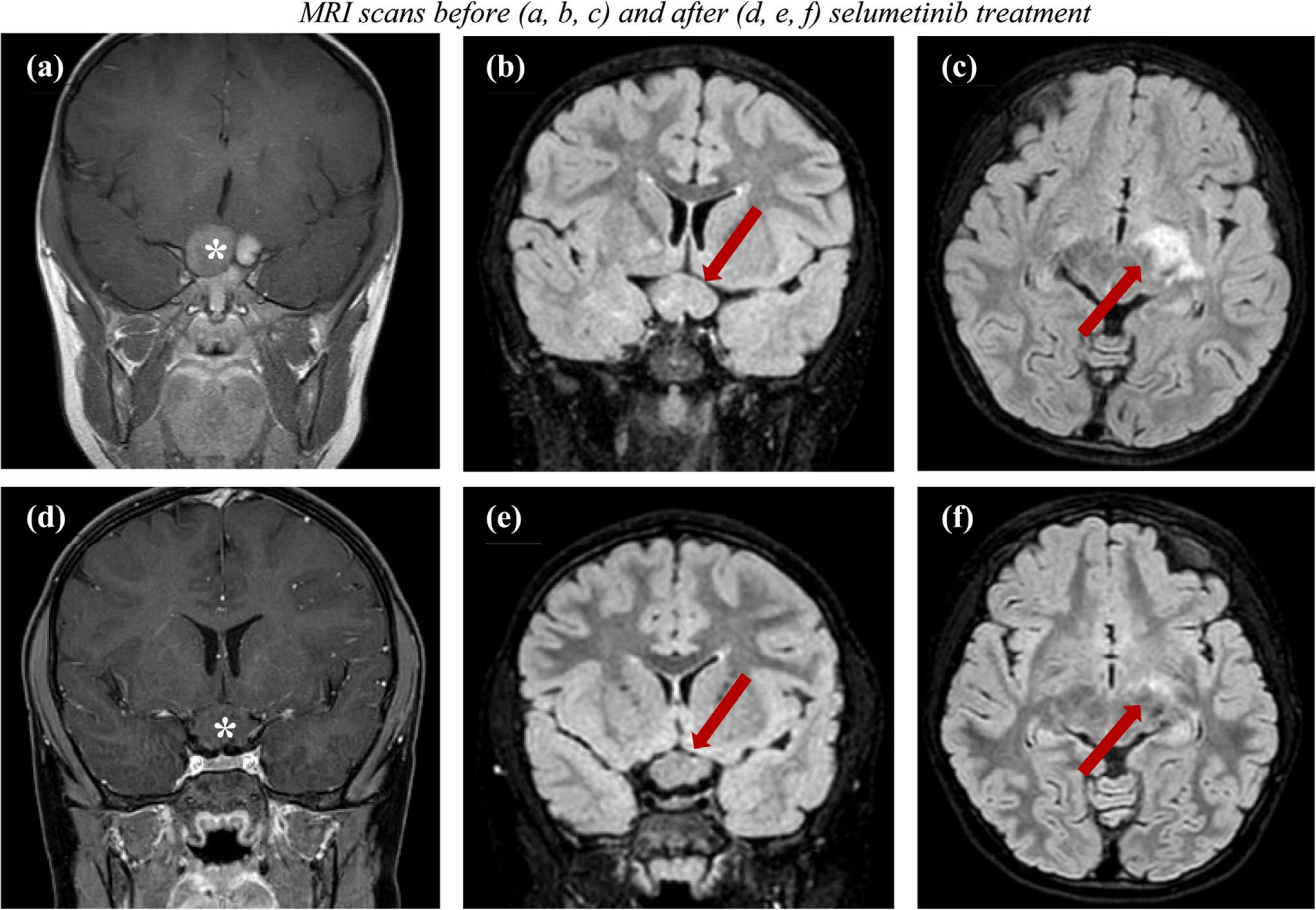
Pre‐selumetinib MRI: (a) coronal post‐contrast T1‐weighted, (b) post‐chemotherapy coronal and axial and (c) axial FLAIR images. Figure (a) demonstrates post‐contrast enhancement of the optic tract lesion; figure (c) shows signal hyperintensity of the optic tract. Two years after starting selumetinib: coronal contrast‐enhanced T1‐weighted (d), coronal FLAIR (e) and axial FLAIR (f) images demonstrate almost complete resolution of the left optic tract FLAIR hyperintensity, along with a slight reduction in the size of the optic nerve glioma and an absence of contrast enhancement.

Case 1 A 10‐year‐old girl was diagnosed with NF1 at age one via genetic testing following multiple café‐au‐lait macules. At age 3, she developed an OPG involving the optic chiasm and right optic nerve, evidenced by RNFL thinning on OCT. Brain MRI revealed a 3 cm enhancing mass at the optic chiasm, extending into the right optic nerve, optic radiations and hypothalamic–pituitary region, compressing the pituitary and carotid siphons (Figure 1a,b). T2/FLAIR hyperintensities were present in the bilateral cerebellum, brainstem and supratentorial white matter.

Given these findings and progressive right eye vision loss, she initially received standard first‐line intravenous carboplatin and vincristine chemotherapy. Severe adverse effects, including vincristine‐induced upper‐limb peripheral neuropathy, high fevers and nausea, necessitated a regimen change to intravenous cisplatin and cyclophosphamide. Despite 3years of chemotherapy, the patient nearly lost all vision in her right eye (Table 1).

**TABLE 1:** Chronological progression of visual acuity changes over the follow‐up period.

	−4 months	−3 months	Treatment	+3 months	+5 months	+13 months	+20 months	+21 months	+35 months
BCVA in OD	1/20	1/20	1/20	1/10	1/10	1/10	1/10	1/10	1/10
BCVA in OS	7/10	5/10	1/10	3/10	4/10	5/10	6.3/10	6–7/10	7/10

Eight months post‐chemotherapy, surveillance MRI showed new tumour growth in the retrochiasmatic region, involving the left optic nerve (Figure 1c,d). Initially, a watchful‐waiting approach was used as left‐eye visual acuity remained stable. However, subsequent imaging revealed glioma enlargement and a decline in left‐eye visual acuity of 1/10 (Table 1). Due to progressive vision loss, salvage weekly vinblastine chemotherapy was suggested.

Due to severe previous toxicities, the family sought alternative treatment at our Institution.

Consequently, off‐label treatment with the oral MEK inhibitor selumetinib (25 mg/m^2^ daily, ~50 mg/day) was initiated in consultation with the Oncology Department.

Pre‐treatment BCVA was 1.3 logMAR (1/20) in the right eye (OD) and 1.0 logMAR (1/10) in the left eye (OS). Orthoptic assessment revealed unsteady fixation, exotropia and lack of stereoacuity. Intraocular pressure (IOP) and biomicroscopy findings from the anterior segment were unremarkable, while fundus examination revealed bilateral optic atrophy. OCT showed a statistically significant reduction in bilateral peripapillary RNFL thickness, worse in OD (49 μm in OD and 59 μm in OS) and VEPs showed a reduced amplitude (2.5 μV with a latency of 151 ms in OD and 7 μV with a latency of 141 ms in OS) of P100 in both eyes (OU), worse in OD. Visual field testing showed bitemporal hemianopia.

In subsequent follow‐ups, an initial stability of the neuroradiologic images was achieved.

Two years into selumetinib treatment, brain MRI revealed a significant reduction in the left‐sided nucleo‐capsular glioma mass and reduced contrast agent uptake in all gliomatous lesions, especially those involving the optic radiations. The cisternal segment of the right optic nerve lesion showed only a slight increase, and the remaining tumour segments were stable (Figure 1e,f).

Thirty‐five months after treatment initiation, BCVA was 1.0 logMAR (1/10) in the OD and 0.12 logMAR (7/10) in the OS; fixation stability had improved, while the rest of the ocular findings remained stable (Table 1). Post‐treatment VEPs showed an improved amplitude of 6 μV with a latency of 135 ms in OD, and an amplitude of 9 μV with a latency of 139 ms in OS. RNFL thickness was recorded at 44 μm in OD and 55 μm in OS.

Selumetinib treatment was well tolerated. Mild dermatologic adverse effects (oral ulcers, eczematous rash) were managed with topical therapy.

Case 2 A 5‐year‐old girl was diagnosed with NF1 at 3 months based on café‐au‐lait macules and confirmed by molecular testing (c.499_502delTGTT frameshift mutation in NF1 exon 4b).

**FIGURE 2:**
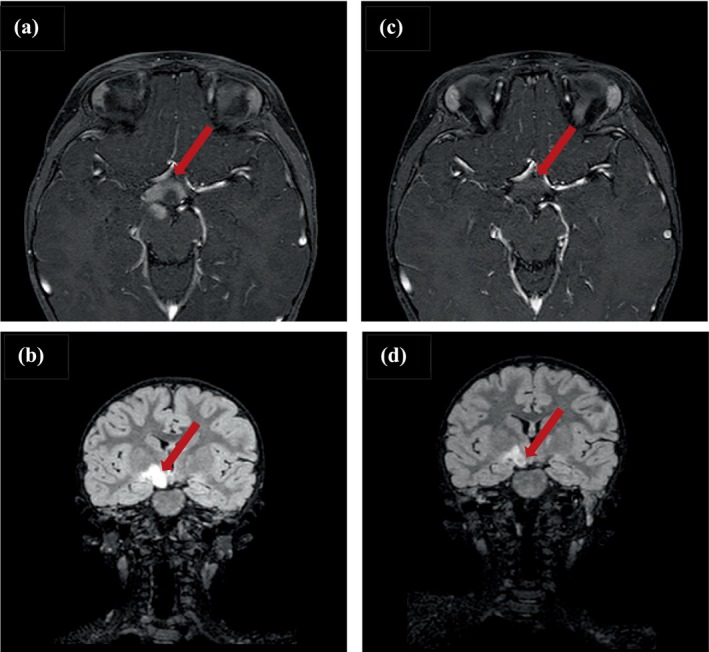
Pre‐therapy MRI (a and b) shows the glioma with contrast enhancement (a) and T2‐FLAIR alterations (b). Post‐selumetinib MRI (c and d) at 4 months displays an almost complete absence of contrast enhancement (c) and a reduction in glioma size (d).

At 3 years of age, a surveillance brain MRI showed bilateral thickening of the infraorbital optic nerves, the prechiasmatic right optic nerve and the optic chiasm. Initially asymptomatic and followed regularly, subsequent imaging revealed dimensional progression with contrast enhancement (Figure 2a,b) and consistently a measurable decline in visual acuity was observed (Table 2).

**TABLE 2:** Longitudinal changes in visual acuity before and after initiation of selumetinib therapy, demonstrating near‐complete vision recovery at nine months.

	−5 months	−3 months	Treatment	+4 months	+12 months	+18 months	+24 months
BCVA in OD	9/10	6/10	4–5/10	5/10	7/10	8/10	10/10
BCVA in OS	10/10	9/10	4–5/10	4–5/10	6/10	8/10	10/10

At 4 years of age, due to neuroradiological progression and worsening vision, standard chemotherapy was recommended elsewhere per guidelines. Concerned about toxicity, the family sought a second opinion at our centre to explore targeted therapy with selumetinib.

At the time of diagnosis, BCVA was 0.05 logMAR (9/10) in OD and 0.00 logMAR (10/10) in OS; orthoptic and ophthalmologic evaluation revealed no abnormalities, with the exception of a Lisch nodule in OU; IOP and visual field testing were normal. OCT revealed an RNFL thickness of 94 μm in OD and 93 μm in OS. VEPs showed an amplitude of 9 μV and latency of 113 ms in OD and an amplitude of 15 μV and latency of 112 ms in OS. During follow‐up, BCVA began to deteriorate, and consistently, VEPs showed a slight increase in P100 latency.

MRI showed a hypothalamic–chiasmatic lesion that spread to the optic radiations, cerebral peduncle and right subthalamic region, along with tortuous, thickened optic nerves and FLAIR hyperintensity (Figure 2).

Considering the entity of the lesion and the progressive deterioration in visual acuity, in agreement with the oncologists, compassionate treatment with selumetinib was started at a dose of 25 mg/m^2^/day, following a multidisciplinary consensus between paediatric oncologists and rare disease specialists, specifically addressing the family's strong desire for a less toxic alternative to conventional chemotherapy.

Four months after starting selumetinib, follow‐up MRI showed a reduced size and T2 FLAIR hyperintensity of the glioma, especially in the chiasmatic region and optic tracts (Figure 2c,d).

Consistently a measurable improvement in BCVA was observed, reaching 0.00 logMAR (10/10) in OU after 24 months of treatment. Post‐treatment VEPs improved, showing an amplitude of 12 μV with a latency of 117 ms in OD, and an amplitude of 18 μV with a latency of 114 ms in OS. RNFL thickness was evaluated at 89 μm in OD and 92 μm in OS. The remaining ophthalmological findings were unchanged (Table 2).

**FIGURE 3:**
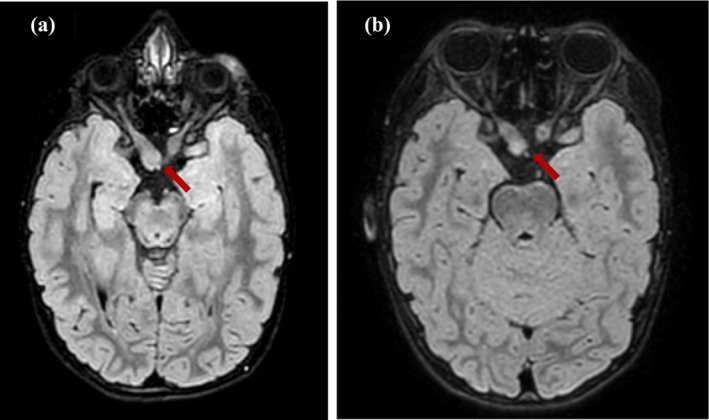
Axial FLAIR MRI (a) before therapy and (b) 1 year and 5 months into selumetinib treatment showed radiological stability (no size increase) despite significant visual improvement. As indicated by the arrow, there are no significant differences at the optic nerve level.

Case 3 A 6‐year‐old boy was diagnosed with NF1 at 6 weeks based on hyperpigmented spots and a family history. At age four, a screening MRI revealed a non‐contrast‐enhancing OPG (11 × 14 mm) in the optic chiasm (right side predominant) with initial involvement of both pre‐chiasmatic optic nerve segments (Figure 3).

At the time of diagnosis, BCVA was 5/10 in OU; orthoptic evaluation, IOP and anterior segment biomicroscopy were unremarkable, while fundus examination revealed mild bilateral optic atrophy.

The lesion was monitored with regular follow‐up, despite apparent MRI stability in size and contrast enhancement, a measurable decline in visual acuity was observed (Table 3). BCVA was 0.7 logMAR (2/10) in OD and 0.4 logMAR (4/10) in OS. Orthoptic evaluation, IOP and anterior segment biomicroscopy were unremarkable, while fundus examination revealed mild bilateral optic atrophy. OCT revealed a statistically significant reduction in bilateral RNFL thickness, worse in OD (54 μm in OD and 76 μm in OS); VEPs showed reduced amplitude and prolonged P100 latency in OU (amplitude 7 μV, latency 127 ms in OD; amplitude 14 μV, latency 119 ms in OS); and visual field testing showed bilateral restriction in the superior visual field.

**TABLE 3:** Evolution of visual acuity over time, highlighting a appreciable recovery during the course of observation.

	−8 months	−6 months	Treatment	+2 months	+5 months	+8 months	+10 months	+18 months
BCVA in OD	4–5/10	4/10	2/10	2–3/10	3/10	6/10	6/10	7/10
BCVA in OS	5/10	4/10	4/10	4/10	5/10	6/10	6/10	9/10

Consequently, off‐label selumetinib (25 mg/m^2^/day) was initiated in agreement with oncologists.

After 1 year and 5 months of therapy, follow‐up MRI showed a slight reduction in optic chiasm swelling, especially in the right portion (up to 11 mm). Pre‐chiasmatic lesions remained stable (Figure 3b), visual acuity gradually improved (Table 3).

Eighteen months after treatment initiation, BCVA was 0.15 logMAR (7/10) in the OD and 0.05 logMAR (9/10) in the OS; the remaining ophthalmological findings were unchanged. Post‐treatment VEP parameters showed an amplitude of 7 μV and latency of 122 ms in OD, and an amplitude of 14 μV and latency of 116 ms in OS. RNFL thickness in OD was measured at 52 μm.

The medication demonstrated good tolerability, with no significant adverse events noted; minor side effects included xerotic skin and the onset of hair depigmentation.

## 4 Results

In all three patients, selumetinib demonstrated both radiological and clinical efficacy. This was evidenced by a significant reduction in tumour volume in two patients and durable radiological stability in the third patient. A particularly notable outcome was the improvement in visual function, which initially stabilized and then showed marked recovery. Specifically, visual acuity substantially improved in all three patients: two experienced bilateral recovery, and one recovered in a single eye.

The treatment was well‐tolerated, with the most common adverse events being mild dermatological issues such as skin eczema, aphthous stomatitis and an acneiform rash. These reactions were successfully managed with topical treatments and never necessitated discontinuing the therapy. Crucially, no major toxicities (gastrointestinal, pulmonary, or cardiological) were observed.

## 5 Discussion

In this small series, selumetinib effectively induced significant tumour regression or stabilization and substantial visual recovery in NF1‐associated OPGs with minimal toxicity, offering favourable tumour control compared to standard chemotherapy.

Selumetinib is a potent, selective, oral targeted therapy already proven effective in NF1‐related neoplasms like plexiform neurofibroma [18]. To our knowledge, this is one of the first reports detailing its ability to reduce tumour mass and restore vision in paediatric NF1‐related OPGs.

We tracked anatomical and functional responses to selumetinib in three children with NF1‐related OPGs using serial MRI, visual acuity, OCT and VEP. All patients achieved durable disease control (stable or reduced tumour burden) for the long term.

Selumetinib's remarkable tumour shrinkage effect was fast, as shown in Case 2, where MRI revealed near‐complete loss of contrast enhancement and significant glioma size reduction just 4 months after starting treatment.

Notably, each patient experienced substantial recovery of vision, whether selumetinib was administered as first‐ or second‐line therapy: two children improved to normal or near‐normal vision, while the third gained several lines of acuity in one eye, significantly reducing functional disability, with confirmed clinical improvement even in the absence of major MRI changes (Case 3).

Case 1 is illustrative, allowing comparison before treatment, after standard chemotherapy and 2 years into selumetinib. Post‐chemotherapy (Panels c, d), coronal and axial FLAIR showed no significant reduction in left optic tract hyperintensity or chiasmal lesion size. Conversely, after 2 years of selumetinib, the glioma significantly regressed, with near‐complete FLAIR hyperintensity resolution (Figure 1e,f), correlating with the patient's major visual improvement.

Despite the inherent limitations of a small retrospective study, our initial experience highlights selumetinib as a promising targeted agent for paediatric NF1‐OPGs. The observed improvements in visual function appear to contrast favourably with the typical outcomes of standard cytotoxic chemotherapy, which primarily aims to preserve residual vision and only rarely leads to visual gains [7], often at the cost of significant therapy‐related toxicity [15]. By comparison, selumetinib demonstrated a markedly more favourable safety profile [24]: in the PBTC phase II trial of selumetinib for NF1‐associated low‐grade gliomas, grade 3–4 toxicities were limited to transient transaminase elevations and rash, with no treatment‐related deaths. It must be noted that standard chemotherapy is similarly not associated with treatment‐related mortality in this setting; furthermore, its discontinuation is most frequently driven by disease progression rather than severe toxicity.

Notably, a large phase III, randomized, multicentre clinical trial (NCT03871257) [25] is currently evaluating selumetinib versus the standard‐of‐care carboplatin and vincristine (CV) regimen in previously untreated, newly diagnosed NF1‐associated LGG. This pivotal study aims to rigorously determine whether selumetinib is non‐inferior to CV in terms of event‐free survival (EFS) and whether it offers superior functional visual outcomes (assessed via visual acuity) in patients with optic pathway involvement.

Furthermore, the landscape of targeted MAPK pathway inhibition for pLGG is rapidly expanding through other similar trials. For instance, the phase III NCT04166409 trial [26] is similarly investigating selumetinib against CV, but in newly diagnosed non‐NF1, non‐BRAF V600E mutant pLGGs. Additionally, other MEK inhibitors are being actively evaluated in this setting: the PLGG‐MEKTRIC phase III trial (NCT05180825, [27]) is currently comparing the MEK inhibitor trametinib to standard weekly vinblastine.

Crucially, mirdametinib, a brain‐penetrant MEK1/2 inhibitor, represents a highly promising future agent that will likely see significant investment for this indication. Preclinical evaluations of mirdametinib in NF1‐mutant low‐grade gliomas have demonstrated notable targeted effects both in vitro using cultured cells and in vivo utilizing a novel zebrafish xenograft model [28].

Translating this robust preclinical rationale to the clinical setting, the Phase I/II SJ901 trial was initiated to evaluate single‐agent mirdametinib for the treatment of children, adolescents and young adults with LGG [29].

Preliminary clinical observations have been highly encouraging, demonstrating that the drug is not only safe and well‐tolerated but also capable of generating meaningful clinical responses.

Together, these rigorously designed trials will be essential to establish evidence‐based guidelines, define the optimal timing and duration of treatment and fully assess the long‐term safety profile of chronic MEK inhibition in the developing child.

The functional improvement observed in our patients points to the promising therapeutic potential of MEK inhibition and could eventually contribute to a paradigm shift in the management of NF1‐related OPG. By directly targeting dysregulation of the Ras/MAPK pathway, selumetinib offers a biologically rational approach that may serve as a clinically advantageous alternative to conventional regimens [2]. Its oral route and sustained tolerability could make it a suitable option for long‐term paediatric treatment, which is crucial for minimizing burden and preserving quality of life.

These preliminary findings suggest selumetinib might be explored as a potential early intervention, not just salvage therapy, for NF1‐OPG, particularly in high‐risk children (visual morbidity or chemotherapy toxicity concerns). MEK inhibition, which in our limited experience showed a capacity to substantially improve visual function in symptomatic OPGs even following prior treatment failure, warrants further investigation.

Observed in both first‐line and salvage settings within our small cohort, this further supports the need for prospective studies to validate this potential paradigm shift in the management of symptomatic OPGs.

The study's strength lies in its rigorous, multimodal ophthalmological assessment, which integrated serial visual acuity, OCT and VEP. This captured anatomical and functional outcomes, showing functional gains that often correlated with or even preceded/exceeded radiological responses. The prolonged observation period also confirmed the durable nature of both visual recovery and tumour control.

Despite the promising short‐to‐medium‐term outcomes, the continuous use of selumetinib in paediatric patients raises critical clinical questions that remain largely unanswered. Currently, there is a lack of standardized guidelines regarding the optimal duration of therapy and when to safely discontinue treatment. A major concern upon treatment cessation is the potential for a ‘rebound effect’, a rapid, paradoxical tumour regrowth or sudden visual decline following the withdrawal of MEK inhibition, a phenomenon that requires careful monitoring: in the phase 1 trial, Dombi et al. noted that one patient experienced tumour growth during a period of drug interruption due to toxicity, and slow tumour regrowth after maximal response was observed in several patients who had required dose reductions, suggesting a dose‐dependent effect of MEK inhibition [30].

Furthermore, because the MAPK pathway is essential for normal physiological growth, the potential late effects of chronic MEK suppression on developing children – such as impacts on skeletal growth, cardiovascular health and long‐term neurocognitive development‐, are not yet fully characterized [30, 31].

This finding strongly underscores the absolute necessity for vigilant, ongoing clinical monitoring throughout the entire treatment course, rather than only during the initial phases. Therefore, future prospective studies with extended follow‐up are essential not only to confirm response durability but also to establish clear stopping rules, identify patients at risk for rebound progression and define the comprehensive long‐term safety profile of these targeted agents in the paediatric population.

In conclusion, while extensive validation is still required, selumetinib represents a highly promising advancement in the future landscape of paediatric neuro‐oncology [32]. Should ongoing and future trials confirm its long‐term safety profile and establish clear therapeutic protocols, this targeted MEK inhibitor could firmly establish itself as a highly effective, practical and significantly less toxic alternative to conventional chemotherapy. By offering a targeted approach that is easier to administer and potentially more capable of preserving or restoring vision, selumetinib has the potential to profoundly improve both the clinical outcomes and the overall quality of life for children with NF1‐associated OPGs.

## Author Contributions

All authors equally contributed to the study.

## Funding

This work was supported by the Italian Ministry of Health, through the contribution given to the Institute for Maternal and Child Health IRCCS Burlo Garofolo, Trieste—Italy.

## Ethics Statement

The study was approved by the Unique Regional Ethics Committee of Friuli Venezia Giulia (CEUR FVG) (Protocol codes: 29713/2020, 5099/2022 and 9327/2022).

## Consent

According to the Research Institute policy, parents of admitted children are requested to sign an informed consent form for the treatment of anonymized data for research purposes.

## Conflicts of Interest

The authors declare no conflicts of interest.

## Data Availability

Data sharing not applicable to this article as no datasets were generated or analysed during the current study.
